# Prevalence of Anxiety, Depression, and Stress among Teachers during the COVID-19 Pandemic: A Rapid Systematic Review with Meta-Analysis

**DOI:** 10.3390/brainsci11091172

**Published:** 2021-09-03

**Authors:** Naiara Ozamiz-Etxebarria, Nahia Idoiaga Mondragon, Juan Bueno-Notivol, María Pérez-Moreno, Javier Santabárbara

**Affiliations:** 1 Department of Developmental and Educational Psychology, University of the Basque Country UPV/EHU, 48940 Leioa, Spain; nahia.idoiaga@ehu.eus; 2 Psychiatry Service, Hospital Universitario Miguel Servet, 50009 Zaragoza, Spain; elecrijuan@hotmail.com; 3 Pharmacy Service, Hospital Universitario Miguel Servet, 50009 Zaragoza, Spain; marpermor159@gmail.com; 4 Department of Microbiology, Pediatrics, Radiology and Public Health, University of Zaragoza, 50009 Zaragoza, Spain; jsantabarbara@unizar.es; 5 Centro de Investigación Biomédica en Red de Salud Mental (CIBERSAM), Ministry of Science and Innovation, 28029 Madrid, Spain; 6 Aragonese Institute of Health Sciences (IIS Aragón), 50009 Zaragoza, Spain

**Keywords:** stress, anxiety, depression, teachers, gender, school, university, countries, COVID-19, meta-analysis

## Abstract

Background: Since the beginning of the COVID-19 pandemic, teachers have been accumulating adverse psychological symptoms due to the closure of educational centers and the need to adapt to different teaching modalities. Methods: Medline and PubMed were searched for studies on the prevalence of depression, anxiety, stress, and burn-out in teachers, published from 1 December 2019 to 15 June 2021. Results: In total, eight studies were included in this study. The results show that teachers report levels of anxiety (17%), depression (19%), and stress (30%). In Asia, there has been more anxiety compared to other continents. Overall, anxiety has been higher among teachers in schools compared to universities. However, stress levels have been higher among teachers in universities compared to schools. Statistically, there were no significant differences regarding gender and age in any of the symptoms. Conclusions: The results suggest that teachers at different educational levels are experiencing adverse psychological symptomatology during the COVID-19 pandemic, and that anxiety levels vary between different countries. However, more international studies are needed to fully understand the impact of the pandemic on teachers’ mental health.

## 1. Introduction

In recent years, teachers have been showing more adverse psychological symptoms and higher sick-leave rates associated with these [1]. The pressure linked to work [2], together with the loss of status [3,4], has meant that stress [5,6], anxiety [7], depression [8], and burnout [9] have become common among teachers in different countries.

It should also be noted that the COVID-19 pandemic has led to significant health, social, psychological, economic, and educational changes around the world [10,11]. Among these changes, the closure of schools and universities has been one of the most widely implemented measure since the beginning of the pandemic to help maintain social distancing and slow down the spread of the virus [12,13]. According to UNESCO (United Nations Educational, Scientific and Cultural Organization), the duration of school closures has varied across countries and regions [14].

Moreover, since the closure of schools, research has shown that teachers have accumulated psychological symptoms, such as stress, anxiety, and depression, worldwide [15,16,17]. In fact, UNESCO [18] has already identified confusion and stress among teachers as one of the adverse consequences of social distancing measures.

This symptomatology is related to different causes. To begin with, it is related to the rapid change from face-to-face to virtual teaching, also known as emergency e-learning. In fact, many experts have pointed out that pandemic e-learning differs greatly from the deliberate and well-designed online teaching and that it has been perceived as an abrupt and unplanned change in learning contexts that has not been chosen by either learners or teachers [19]. Furthermore, in this context, the organization and didactic planning to transfer teaching contents into an online environment while maintaining their relevance has been a great challenge for teachers of all academic levels [20,21].

Likewise, the emergency e-learning has also provoked changes in teachers’ workload [22], at least in six motivational characteristics of the academic teachers’ job (task identity, task significance, skill variety, feedback, autonomy, and social dimensions of the work). All of these changes have impacted teachers’ motivation towards their own work [23]. Consequently, some of the research conducted focusing on teachers during the period of school closures and lockdown suggests that this crisis has caused symptoms such as anxiety or depression in teachers, in addition to increased rates of divorce and domestic violence, all of which may limit their ability to teach adequately [24].

Furthermore, teachers, especially in early childhood and primary education, are a highly feminized group. Consequently, there is a high number of female teachers who, during the lockdown period, had to take on the burden of caring responsibilities (children, elderly people, etc.) at home and combine these with their profession [5]. This is why female teachers may have had more stress, anxiety, and depression than men in the context of the pandemic [25,26].

However, the opening of schools and the return to classes did not make the psychological symptomatology among teachers disappear [27,28]. Indeed, the reopening of schools itself, amidst great uncertainty and controversy in many countries over the pandemic’s development, was a stressful time for many teachers [17,29,30].

In addition, when classes were reactivated, teachers had to prevent the spread of the virus and deal with selective lockdown and restrictive measures while performing their teaching activities [31]. In fact, the measures imposed to prevent contagion in schools have also had a direct impact on the way that teaching has been carried out, with many classes taking the bimodal or hybrid format (half of the students at home and half in class) [32,33], mirror classrooms [34], small bubble groups [35], or even going online for the whole or part of the 2020/2021 school year [36]. It should also be remembered that the protocols of the educational centers varied significantly between countries or even regions within the same country [14].

In terms of age, since the beginning of the pandemic, it has been observed that, among the general population, it is the younger people who suffered more psychological symptoms, such as anxiety, depression, specific phobias, cognitive change, avoidance, and compulsive behavior [37]. However, some studies with a focus on teachers have shown more psychological symptomatology in older people [17], and this may be due to the digital gap [38].

In regard to the teaching professional sector, in the current pandemic, teachers feel a greater responsibility for younger children, as they need more care and protection. This may lead teachers to feel under pressure to provide adequate care for children, in addition to addressing the concerns of their families [39]. Secondary-school and university teachers, however, may have felt less pressure in this respect, as their students are more autonomous and do not require such care from teachers [40].

Regarding the COVID-19 impact in different countries, the pandemic has impacted education in all countries. However, as UNESCO [14] points out, there have been differences in the conditions and measures implemented in education in different countries. When the pandemic began and schools were closed, only half of the countries with closed schools had alternatives to continue delivering teaching and learning [41]. According to UNESCO [42], the majority of countries that made education alternatives available were from Eastern Europe and Central Asia, followed by Asia and the Pacific, and finally Western Europe and North America. Therefore, inequalities in education in different countries [43] may also bring different psychological symptomatology among teachers.

It is therefore obvious that the pandemic has affected the psychological state of teachers; however, to our knowledge, there has been no systematic review and meta-analysis that has analyzed it. Therefore, the current meta-analysis aims to update the existing evidence on the prevalence of stress, anxiety, and depression among teachers during the COVID-19 pandemic. More specifically, it is intended to analyze whether gender, age range, country, and the academic sector affect the symptomatology of these professionals.

## 2. Materials and Methods

This study was conducted in accordance with the PRISMA (Preferred Reporting Items for Systematic Reviews and Meta-Analyses) guidelines for reporting systematic reviews and meta-analysis [44] (Supplementary Materials Table S1).

### 2.1. Search Strategy

Two researchers (JBN and MPM) searched for all cross-sectional studies reporting the prevalence of depression, anxiety, stress, and burnout published from 1 December 2019 through 15 June 2021, using MEDLINE via PubMed. The search proceeded as follows:

“(“School Teachers”[Mesh] OR “Faculty”[Mesh] OR teacher*[tiab] OR professor*[tiab] OR lecturer*[tiab] OR instructor*[tiab]) AND (Depression[Mesh] OR Depressive Disorder[Mesh] OR depress*[tiab] OR Anxiety[Mesh] OR Anxiety Disorders[Mesh] OR anxi*[tiab] OR Trauma and Stressor Related Disorders[Mesh] OR “Stress, Psychological”[Mesh] OR stress*[tiab] OR Burnout, Psychological[Mesh] OR burnout[tiab])”.

No language restriction was made. References from selected articles were inspected to detect additional potential studies. Then we performed a manual search of the “grey literature” (e.g., medRxiv or Google Scholar) to detect other potentially eligible investigations. Any disagreement was resolved by consensus among third and fourth reviewers (NO-E and NI).

### 2.2. Selection Criteria

Studies were included if (1) reported cross-sectional data on the prevalence of depression, anxiety, stress, or burnout, or sufficient information to compute this, conducted during the COVID-19 outbreak; (2) focused on teachers; (3) included a validated instrument to assess the above outcomes; and (4) the full text was available.

We excluded studies focusing only on community-based samples of general population or specific samples that were not teachers (e.g., students, medical professionals, and patients), as well as review articles.

A predesigned data-extraction form was used to extract the following information: country, sample size, proportion of women, average age, response rate, and sampling methods, and also the instruments used to assess outcomes and their prevalent rates.

### 2.3. Methodological Quality Assessment

Articles selected for retrieval were assessed by two independent reviewers (JBN and JS) for methodological validity before they were included in the review using the Joanna Briggs Institute (JBI) standardized critical appraisal instrument for prevalence studies [45]. Quality was evaluated according to nine criteria, with each yielding a score of zero or one. One score was obtained for each criterion if the study was affirmative in the next questions: (1) Was the sample frame appropriate to address the target population? (2) Were study participants recruited in an appropriate way? (3) Was the sample size adequate? (4) Were the study subjects and setting described in detail? (5) Was data analysis conducted with sufficient coverage of the identified sample? (6) Were valid methods used for the identification of the condition? (7) Was the condition measured in a standard, reliable way for all participants? (8) Was there appropriate an statistical analysis? (9) Was the response rate adequate, and if not, was the low response rate managed appropriately?

Any disagreements that arose between the reviewers were resolved through discussions, or by further discussion with a third and fourth reviewers (NO-E and NI).

### 2.4. Data Extraction and Statistical Analysis

Freeman and Tukey’s double arcsine transformation of prevalence to stabilize the variance was applied [46]. A generic inverse variance method with a random effect model was used [47], which is more appropriate than fixed-effect models when the number of studies included in the meta-analysis is low (<10) [48]. The Hedges Q statistic was reported to check heterogeneity across studies, with statistical significance set at *p* < 0.10. The *I*^2^ statistic and 95% confidence interval (95% CI) were also used to quantify heterogeneity [49]. Values between 25% and 50% are considered low, between 50% and 75% are moderate, and 75% or more are high [50]. Heterogeneity of effects between studies occurs when differences in results for the same exposure-disease association cannot be fully explained by sampling variation. Sources of heterogeneity can include differences in study design or in demographic characteristics. We performed subgroup analyses to explore the sources of heterogeneity expected in meta-analyses of observational studies [51]. Meta-regression was not performed, due to lack of statistical power with less than 10 studies included in a meta-analysis [52]. We conducted a sensitivity analysis to determine the influence of each individual study on the overall result by omitting studies one by one. Publication bias was determined through visual inspection of a funnel plot and also Egger’s test [53] (*p*-values < 0.05 indicate publication bias), since funnel plots were found to be an inaccurate method for assessing publication bias in meta-analyses of proportion studies [54].

Statistical analyses were conducted by using JS and run with STATA statistical software (version 10.0; College Station, TX, USA) and R [55].

## 3. Results

**Figure 1:**
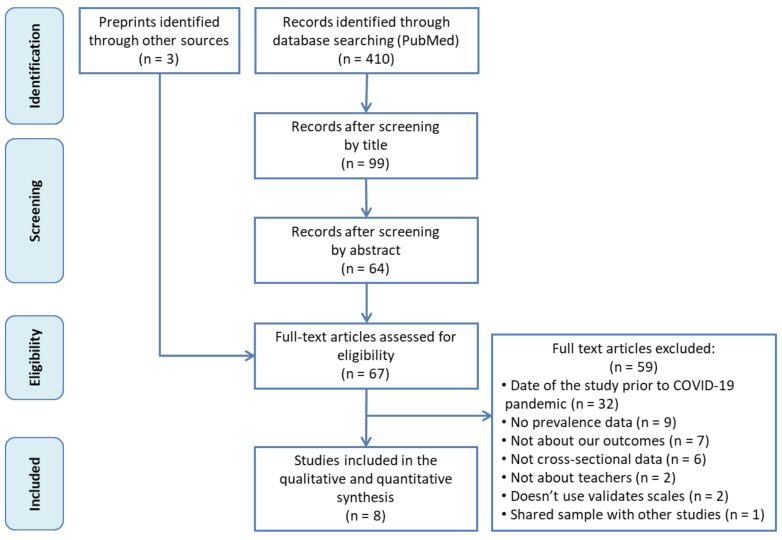
Flowchart of the study search and selection process.

Figure 1 shows the flowchart of the search strategy and study selection process. A total of 410 records were initially identified from Medline via PubMed, and 346 were excluded after a first screening of the titles and abstracts. Three extra records were then added after a manual search in a preprints database (MedRxiv). After reading the remaining 67 articles in full, we finally included eight in our meta-analysis [15,16,17,31,56,57,58,59]. Exclusion reasons are detailed in Figure 1.

Table 1 and Table 2 show the characteristics of the nine studies included in our meta-analysis. Table 1 gives a descriptive overview of the global characteristics, while Table 2 breaks down the methods of measurement of the primary outcomes and the prevalence found in each study. Six studies measured anxiety, five measured stress, and three measured depression levels. For this, all studies used standardized and validated scales, with the most widely used scale being the Depression, Anxiety, and Stress Scale (DASS, n = 3 studies). No articles were found that provided data on professional burnout in teachers during the Covid-19 pandemic.

The sample size ranged from 100 to 88,611 participants, and the mean age ranged from 31.4 to 43.9 years in the five reporting studies. All studies included both men and women, and the percentage of women ranged from 32% to 80%. All studies were conducted by using online questionnaires, and, of those reporting sampling methodology, all used non-randomized methods. Five studies reported the response rate, which ranged from 11% to 99%.

The risk of bias scores ranged from five to eight out of a possible total of nine, with a mean score of 6.6 (SD = 1.2) (Table 3). The main limitation (a), present in all studies, was that the recruitment of participants was inadequate, as all used non-randomized techniques or did not report the method in this regard. The other most common limitations were (b) response rate not reported, or large number of non-responders (six studies), and (c) sample size too small to ensure good precision of the final estimate (three studies).

### 3.1. Prevalence of Anxiety

**Figure 2:**
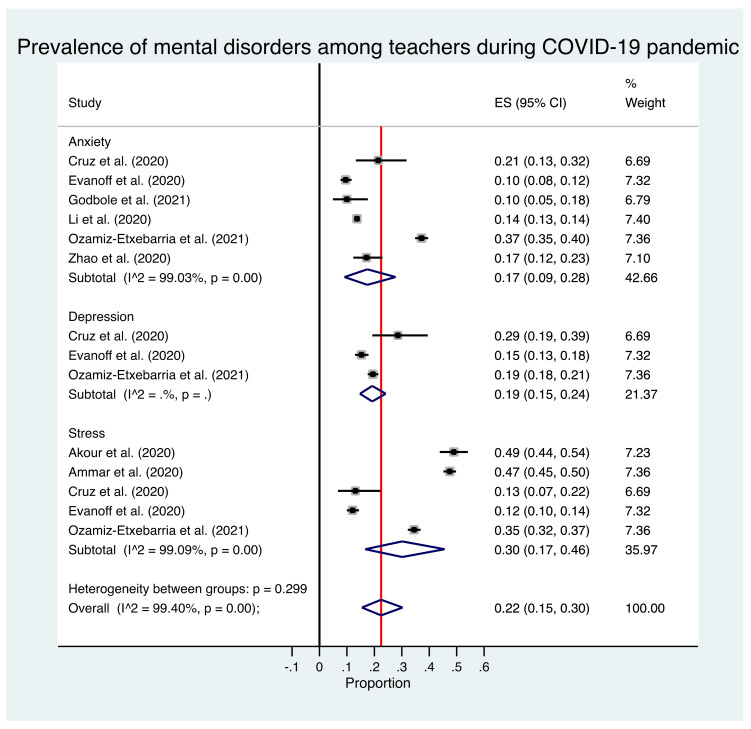
Forest plot for the prevalence of mental disorders among teachers.

**Figure 3:**
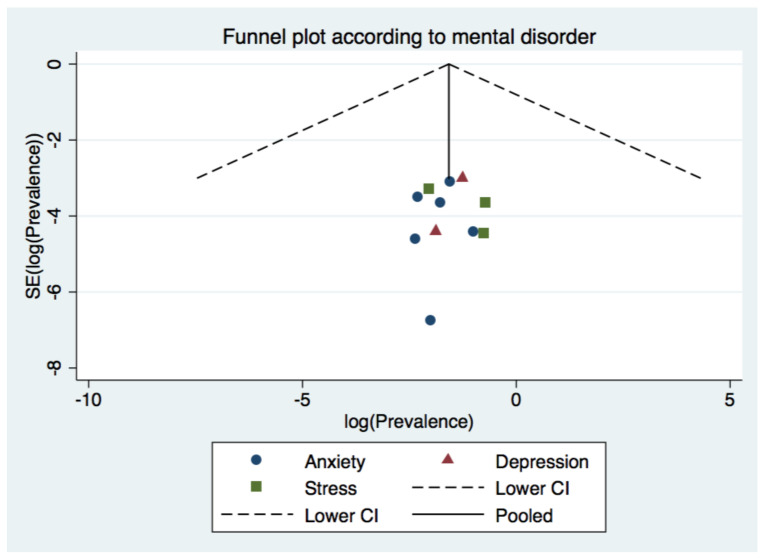
Funnel plot for the prevalence of mental disorders.

The estimated overall prevalence of anxiety was 17% in teachers (95% CI: 9–28%), with significant heterogeneity between studies (Q test: *p* < 0.001; *I*^2^ = 99%) (Figure 2). Lower prevalence of anxiety was found for studies located in Asia (14% [95% CI: 11–16%]) compared to those located in other continents (22% [95% CI: 5–46%]); however, this difference did not reach statistical significance. In particular, studies conducted in China (14% [95% CI: 13–14%]) showed a lower prevalence of anxiety compared with studies conducted in other countries (19% [95% CI: 5–38%]). We also observed higher prevalence of anxiety for studies using the DASS-21 (22% [95% CI: 5–46%]) compared to those using the HAM-A/GAD-7/SAS (14% [95% CI: 11–16%]), and those focused in school teachers (16% [95% CI: 10–22%]) compared with studies focused in University teachers (10% [95% CI: 8–12%]); however, this difference did not reach statistical significance. No subgroup analysis according to sampling method was performed due to insufficient data available. Excluding each study one by one from the analysis did not substantially change the pooled prevalence of anxiety, which varied between 13% (95% CI: 10–17%), with Ozamiz-Etxebarria et al. excluded, and 18% (95% CI: 6–34%), with Li et al. excluded. This indicates that no single study had a disproportional impact on the overall prevalence. Visual inspection of the funnel plot (Figure 3) suggested no publication bias presence for the estimate of prevalence of anxiety in teachers, confirmed by non-significant results from the Egger’s test (*p* = 0.856).

### 3.2. Prevalence of Depression

Only tree studies reported prevalence of depression data. The estimated overall prevalence of depression was 19% in teachers (95% CI: 15–24%), with significant heterogeneity between studies (Q test: *p* < 0.001; *I*^2^ = 83.7%) (Figure 2). No subgroups analyses were performed due to insufficient number of studies available. Visual inspection of the funnel plot (Figure 3) suggested no publication bias presence for the estimate of prevalence of anxiety in teachers, confirmed by non-significant results from the Egger’s test (*p* = 0.263).

### 3.3. Prevalence of Stress

The estimated overall prevalence of stress was 30% in teachers (95% CI: 17–46%), with significant heterogeneity between studies (Q test: *p* < 0.001; *I*^2^ = 99.1%) (Figure 2). A statistically significant higher prevalence of anxiety for studies using the DASS-21 (19% [95% CI: 6–38%]) was observed compared to those using the K10 or IES (48% [95% CI: 46–50%]). We also observed lower prevalence of stress in school teachers (13% [95% CI: 7–22%]) compared with studies focused in University teachers (35% [95% CI: 12–66%]); however, this difference did not reach statistical significance. Studies using the snowball sampling method report a lower prevalence of stress (37% [95% CI: 35–39%]) compared to those with convenience sampling method (47% [95% CI: 45–50%]), with this difference being statistically significant. No subgroup analysis according to geographical location was performed due to insufficient data available. Excluding each study one by one from the analysis did not substantially change the pooled prevalence of anxiety, which varied between 26% (95% CI: 11–44%), with Ammar et al. excluded, and 36% (95% CI: 27–47%), with Evanoff et al. excluded. This indicates that no single study had a disproportional impact on the overall prevalence.

Our visual inspection of the funnel plot (Figure 3) suggested no publication bias presence for the estimate of prevalence of anxiety in teachers, confirmed by non-significant results from the Egger’s test (*p* = 0.648).

## 4. Discussion

### 4.1. Summary of Main Findings

The COVID-19 pandemic is having an unprecedented impact on teachers, with stress, anxiety, and depression being the most reported mental symptomatology. The present study provides an updated meta-analysis of studies reporting on the prevalence of stress, anxiety, and depression among teachers during the COVID-19 pandemic. Our meta-analysis is based on eight studies, and, to the best of our knowledge, this is the first review to report on overall prevalence rates of stress, anxiety, and depression across different ages, gender, countries, and educational sectors in teachers. Our findings show that teachers report levels of anxiety (17%), depression (19%), and stress (30%) with significant heterogeneity among the reviewed studies. These results were somewhat higher (especially referring to stress) than those recently found (in 2021) in a meta-analysis conducted in the general population during the pandemic where the prevalence of anxiety was 15.5%, the prevalence of depression was 15.97%, and the prevalence of stress was 13.29% [60]. Some of the research carried out on this subject has highlighted that this symptomatology may be due to emergency e-learning, teachers’ overload [22] and the uncertainty about the reopening of schools in the midst of the pandemic [28].

In terms of gender, there were no significant differences between male and female in neither stress or anxiety, and studies measuring depression did not differ on this variable. This finding is opposed to the initial expectations, as studies among the general population suggest that females are suffering more psychological symptoms during this pandemic [61]. Moreover, it was also expected that female teachers would have more symptoms than men in the context of the pandemic [25,26] due to the burden of caring responsibilities at home combined with their profession [5]. This may be due to the fact that teaching staff in general is composed of females, and therefore there may not have been significant results in terms of gender because of the feminization of the profession [62].

There were also no age differences among the symptomatology, despite the fact that older teachers were expected to have more symptomatology due to difficulties in adapting to the new emergency e-learning system [38].

Regarding country differences, a lower prevalence of anxiety was found in the studies located in Asia, in particular, the studies conducted in China. In a cross-country research performed to find differences in anxiety and behavioral response to the COVID-19 pandemic, it was found that anxiety was less common among patient societies, such as Asian societies [63]. In addition, teachers in Asia were already more familiar with e-learning and may have had more technological resources compared to other countries [64] and therefore may not have reported as much anxiety compared to other countries where technological resources might have been limited [42].

Finally, the most surprising finding is that higher anxiety levels were found at elementary levels of education, as expected, but more teachers with stress were found among university teachers. It may be that there was more anxiety among school teachers as this symptom is an emotional reaction of alertness to a threat [65]. Therefore, the threat of the pandemic was a one-off event among school teachers, as many of them had to work with groups of children without masks or have direct contact with them. However, stress is a broader process of adaptation to the environment, and it is well-known that university teachers have been accumulating stress long before the pandemic [66]. University teachers are responsible for the important task of training students in a variety of advanced specialized skills and promoting the development of science and technology and social progress, which are fundamental to any country’s prosperity [67]. However, all of these tasks may create stress symptomatology, in particular, at a time of uncertainty and high workload, such as during this pandemic [68]. In addition, university teachers must constantly interact with students, maintain a high level of professional performance, and meet targets and deadlines, even in times of pandemic. Thus, these are all factors that may increase stress among this group [69]. In addition, some “old diseases” that continue to exist among people not affected by COVID-19 should not be forgotten [70,71]. Pathologies such as neurosurgical, neurological, and psychiatric, among others, of the population have been blocked due to the COVID-19 pandemic, and this may have increased the emotional burden among people suffering from these diseases [71].

Therefore, considering the findings of the present work, it is necessary to reduce the psychological impact and to improve and avoid these situations of stress, anxiety, and depression during the pandemic among teachers. Having psychologically healthy teachers will be useful to avoid job losses due to emotional distress and will improve the quality of education for students. Therefore, it would be important that they receive support in the form of additional teachers and resources. It would also be important for them to receive emotional support by introducing workshops to strengthen the emotional resources of teachers in schools. In this way, the emotional environment in schools could be improved and the mental health of teachers could be protected. This improvement would have an impact on the mental health of pupils and their academic performance.

### 4.2. Strengths and Limitations

The greatest strength of the present study is that, to our knowledge, no meta-analysis has been carried out that focuses on teachers’ symptomatology in the face of the pandemic. This is why this study may provide the basis for further studies along this research line. Moreover, a rigorous approach to identify publication bias has been implemented (i.e., Egger’s test) which has demonstrated that there is no bias in the estimation of the pooled prevalence of anxiety, stress, and depression for teachers.

However, some limitations should be considered when interpreting our results, due to the biases presented in the grouped estimation of the results. One of the major limitations of the study is the quality of the available literature. Since the systematic review requires previous existing scientific publications, when evaluating any condition during the pandemic using this methodology, there will be a scarce availability of information and a high risk of including literature of moderate-to-low methodological quality. In the same vein, there have not yet been many studies on teachers’ symptomatology conducted in the face of the pandemic. In particular, no study was found that measured burnout, although this symptomatology was widely found in teaching before the pandemic [9].

Furthermore, the majority of the research reviewed was based on cross-sectional data and non-probabilistic samples and used a variety of self-report scales. Indeed, the studies that use DASS are non-Asian studies, and this is why the results are repeated. Therefore, as the epidemiological status of COVID-19 is constantly changing worldwide, longitudinal studies would be necessary to determine whether the elevated levels of anxiety, stress, and depression are sustained, reduced, or increased over time [72].

## 5. Conclusions

This meta-analysis shows that the proportion of teachers suffering from anxiety, stress, or depression during the COVID-19 pandemic is considerable. Therefore, there is an urgent need to prevent and treat common mental health issues among this population cohort. Specifically, the data show that the difference in measures implemented to deal with the impacts of the pandemic in education between countries is worrying, and that it is necessary to support those countries that may be facing greater challenges. In fact, there is already a large gap between countries in terms of progress in dealing with the pandemic [73,74], and it is important to address these inequalities, as these may be impacting essential social pillars, such as education.

It is also important to pay attention to the different symptomatology that teachers may be experiencing at different educational levels and provide the necessary resources to deal with these symptoms. Improving the emotional state of teachers would have a direct impact on their students, as it directly influences the quality of education and the emotional state of students [75].

## Figures and Tables

**Table 1:** Characteristics of the studies included in the meta-analysis.

First Author (Publication Year)	Sample Country	Population	Sample Size (n)	Mean Age (SD)	% Females (n)	Response Rate (%)	Sampling Method	Quality Assessment
Akour et al. (2020)	Jordan	University teachers	382	43.9 (9.9)	44.50% (170)	98.7%	Snowball	8
Ammar et al. (2020)	Global	University teachers	1862	NR	53.47% (996)	11.3%	Convenience	7
Cruz et al. (2020)	Brazil	School teachers	84	31.4 (NR)	75.00% (63)	80.7%	NR	5
Evanoff et al. (2020)	USA	University teachers	870	NR	60.11% (523)	19.7%	NR	7
Godbole et al. (2021)	India	School teachers	100	38.7 (NR)	32.00% (32)	NR	Convenience	5
Li et al. (2020)	China	All teachers	88611	36.22 (9.02)	76.93% (68169)	94.8%	Cluster	8
Ozamiz-Etxebarria et al. (2021)	Spain	All teachers	1623	42.6 (9.96)	79.67% (1293)	NR	Snowball	7
Zhao et al. (2020)	China	School teachers	210	NR	79.19% (160)	NR	NR	6

Abbreviations: SD, standard deviation; NR, not reported.

**Table 2:** Outcome assessments of the included studies.

First Author (Publication Year)	Anxiety Assessment			Depression Assessment			Distress Assessment		
	Scale	Criteria	Prevalence	Scale	Criteria	Prevalence	Scale	Criteria	Prevalence
Akour et al. (2020)							K10	≥25	187 (49%)
Ammar et al. (2020)							IES	≥26	884 (47.5%)
Cruz et al. (2020)	DASS-21	NR	18 (21.4%)	DASS-21	NR	24 (28.6%)	DASS-21	NR	11 (13.1%)
Evanoff et al. (2020)	DASS-21	Moderate to high	83 (9.5%)	DASS-21	Moderate to high	133 (15.3%)	DASS-21	Moderate to high	105 (12.1%)
Godbole et al. (2021)	HAM-A	≥18	10 (10%)						
Li et al. (2020)	GAD-7	≥10	12110 (13.7%)						
Ozamiz-Etxebarria et al. (2021)	DASS-21	Moderate to high	604 (37.2%)	DASS-21	Moderate to high	316 (19.5%)	DASS-21	Moderate to high	560 (34.5%)
Zhao et al. (2020)	SAS	≥50	36 (17.1%)						

Abbreviations: DASS-21, Depression, Anxiety, and Stress Scale; GAD-7, Generalized Anxiety Disorder scale; HAM-A, Hamilton Rating Scale for Anxiety; IES, Impact of Event Scale; K10, Kessler Psychological Distress Scale; NR, not reported; SAS, Zung Self-Rating Anxiety Scale.

**Table 3:** Quality assessment.

Study	1	2	3	4	5	6	7	8	9	TOTAL
Akour et al. (2020)	Y	N	Y	Y	Y	Y	Y	Y	Y	8
Ammar et al. (2020)	Y	N	Y	Y	Y	Y	Y	Y	N	7
Cruz et al. (2020)	N	U	N	Y	Y	Y	Y	Y	U	5
Evanoff et al. (2020)	Y	U	Y	Y	Y	Y	Y	Y	N	7
Godbole et al. (2021)	N	N	N	Y	Y	Y	Y	Y	U	5
Li et al. (2020)	Y	N	Y	Y	Y	Y	Y	Y	Y	8
Ozamiz-Etxebarria et al. (2021)	Y	N	Y	Y	Y	Y	Y	Y	N	7
Zhao et al. (2020)	Y	U	N	Y	Y	Y	Y	Y	U	6

Abbreviations: N, no; Y, yes, U, unclear; (1) Was the sample frame appropriate to address the target population? (2) Were study participants recruited in an appropriate way? (3) Was the sample size adequate? (4) Were the study subjects and setting described in detail? (5) Was data analysis conducted with sufficient coverage of the identified sample? (6) Were valid methods used for the identification of the condition? (7) Was the condition measured in a standard, reliable way for all participants? (8) Was there an appropriate statistical analysis? (9) Was the response rate adequate, and if not, was the low response rate managed appropriately?

## Data Availability

Not applicable.

## Supplementary Materials

The following are available online at https://www.mdpi.com/article/10.3390/brainsci11091172/s1. Table S1: PRISMA Checklist.
